# Vitamin D and Risk of Multiple Sclerosis: A Mendelian Randomization Study

**DOI:** 10.1371/journal.pmed.1001866

**Published:** 2015-08-25

**Authors:** Lauren E. Mokry, Stephanie Ross, Omar S. Ahmad, Vincenzo Forgetta, George Davey Smith, Aaron Leong, Celia M. T. Greenwood, George Thanassoulis, J. Brent Richards

**Affiliations:** 1 Centre for Clinical Epidemiology, Department of Epidemiology, Lady Davis Institute for Medical Research, Jewish General Hospital, McGill University, Montreal, Quebec, Canada; 2 Department of Medicine, McGill University, Montreal, Quebec, Canada; 3 MRC Integrative Epidemiology Unit, School of Social and Community Medicine, University of Bristol, Bristol, United Kingdom; 4 Division of General Internal Medicine, Massachusetts General Hospital, Boston, Massachusetts, United States of America; 5 Department of Medicine, Harvard Medical School, Boston, Massachusetts, United States of America; 6 Department of Oncology, McGill University, Montreal, Quebec, Canada; 7 Department of Epidemiology, Biostatistics and Occupational Health, McGill University, Montreal, Quebec, Canada; 8 Department of Human Genetics, McGill University, Montreal, Quebec, Canada; 9 Lady Davis Institute, Jewish General Hospital, Montreal, Quebec, Canada; 10 Preventive and Genomic Cardiology, McGill University Health Center, Montreal, QC; 11 Department of Twin Research and Genetic Epidemiology, King’s College London, United Kingdom; Imperial College London, UNITED KINGDOM

## Abstract

**Background:**

Observational studies have demonstrated an association between decreased vitamin D level and risk of multiple sclerosis (MS); however, it remains unclear whether this relationship is causal. We undertook a Mendelian randomization (MR) study to evaluate whether genetically lowered vitamin D level influences the risk of MS.

**Methods and Findings:**

We identified single nucleotide polymorphisms (SNPs) associated with 25-hydroxyvitamin D (25OHD) level from SUNLIGHT, the largest (*n* = 33,996) genome-wide association study to date for vitamin D. Four SNPs were genome-wide significant for 25OHD level (*p*-values ranging from 6 × 10^−10^ to 2 × 10^−109^), and all four SNPs lay in, or near, genes strongly implicated in separate mechanisms influencing 25OHD. We then ascertained their effect on 25OHD level in 2,347 participants from a population-based cohort, the Canadian Multicentre Osteoporosis Study, and tested the extent to which the 25OHD-decreasing alleles explained variation in 25OHD level. We found that the count of 25OHD-decreasing alleles across these four SNPs was strongly associated with lower 25OHD level (*n* = 2,347, F-test statistic = 49.7, *p* = 2.4 × 10^−12^). Next, we conducted an MR study to describe the effect of genetically lowered 25OHD on the odds of MS in the International Multiple Sclerosis Genetics Consortium study, the largest genetic association study to date for MS (including up to 14,498 cases and 24,091 healthy controls). Alleles were weighted by their relative effect on 25OHD level, and sensitivity analyses were performed to test MR assumptions. MR analyses found that each genetically determined one-standard-deviation decrease in log-transformed 25OHD level conferred a 2.0-fold increase in the odds of MS (95% CI: 1.7–2.5; *p* = 7.7 × 10^−12^; *I* ^2^ = 63%, 95% CI: 0%–88%). This result persisted in sensitivity analyses excluding SNPs possibly influenced by population stratification or pleiotropy (odds ratio [OR] = 1.7, 95% CI: 1.3–2.2; *p* = 2.3 × 10^−5^; *I* ^2^ = 47%, 95% CI: 0%–85%) and including only SNPs involved in 25OHD synthesis or metabolism (OR_synthesis_ = 2.1, 95% CI: 1.6–2.6, *p* = 1 × 10^−9^; OR_metabolism_ = 1.9, 95% CI: 1.3–2.7, *p* = 0.002). While these sensitivity analyses decreased the possibility that pleiotropy may have biased the results, residual pleiotropy is difficult to exclude entirely.

**Conclusions:**

A genetically lowered 25OHD level is strongly associated with increased susceptibility to MS. Whether vitamin D sufficiency can delay, or prevent, MS onset merits further investigation in long-term randomized controlled trials.

## Abstract 2

J. Brent Richards and colleagues assess whether genetically predicted vitamin D levels associate with risk of multiple sclerosis.

## Editors' Summary

**Background:**

Multiple sclerosis (MS) is a debilitating neurological disorder that affects the nerves in the brain and spinal cord and that usually presents between the ages of 20 and 40 years. It is an autoimmune disease—a disease in which the body’s immune system attacks healthy tissues. In the case of MS, the immune system attacks myelin, the fatty tissue that forms an insulating sheath around nerves fibers. Multiple areas of scarring (sclerosis) in the myelin are produced by immune attack, and the damage slows down or blocks the transmission of electrical signals along the nerves to and from the brain and causes symptoms such as loss of vision, muscle stiffness, and daytime fatigue. For most affected individuals, MS initially follows a relapsing–remitting course characterized by periods lasting days or weeks during which symptoms flare up, followed by periods during which symptoms becomes milder or disappear. Half of people with relapsing–remitting MS eventually develop secondary progressive MS, in which their symptoms inexorably worsen. There is currently no cure for MS, but some treatments can relieve its symptoms or reduce the number of relapses.

**Why Was This Study Done?:**

Why the immune system attacks myelin to cause MS is unclear but probably involves both genetic and environmental risk factors. One potential environmental risk factor is reduced levels of vitamin D. Circulating levels of 25-hydroxyvitamin D (25OHD; the clinical determinant of vitamin D status) are determined in part by exposure to sunlight, and MS is more common at higher latitudes, where exposure to sunlight is decreased. Other epidemiological studies (investigations that examine disease patterns in populations) also suggest an association between lower vitamin D level and an increased risk of MS but cannot prove that a decreased vitamin D level actually causes MS. Individuals who develop MS might share another unknown characteristic that increases their risk of MS (confounding), or individuals who have MS might spend less time outdoors and, as a result, have lower circulating vitamin D levels (reverse causation). It is important to know whether a decreased vitamin D level increases the risk of MS because vitamin D insufficiency is becoming increasingly common. Here, the researchers undertake a Mendelian randomization study to determine whether circulating vitamin D level has a causal effect on MS susceptibility. Because gene variants are inherited randomly, they are not prone to confounding. Reverse causation is also prevented since MS does not change genetic variants. So, if vitamin D level actually affects MS risk, genetic variants that affect vitamin D level should be associated with altered susceptibility to MS.

**What Did the Researchers Do and Find?:**

The researchers first ascertained the effect on 25OHD level among participants in the Canadian Multicentre Osteoporosis Study of four single nucleotide polymorphisms (SNPs, a type of genetic variant) that were associated with 25OHD level in a genome-wide association study (SUNLIGHT). Each of the SNPs explained an important proportion of the population-level variance in 25OHD level in the Canadian Multicentre Osteoporosis Study. The researchers then used the SNPs to examine whether there was an association between genetically reduced 25OHD level and susceptibility to MS among participants in the International Multiple Sclerosis Genetics Consortium study, a genome-wide association study involving up to 14,498 people with MS and 24,091 healthy controls. They found that a genetic decrease in the natural-log-transformed 25OHD level by one standard deviation was associated with a 2-fold increased risk of MS. In practical terms, this finding means that increasing an individual’s circulating 25OHD level by approximately 1.5-fold decreases their odds of developing MS by 50%.

**What Do These Findings Mean?:**

These findings show that, among the participants of the International Multiple Sclerosis Genetics Consortium study, all of whom were of European ancestry, genetically lowered 25OHD level was strongly associated with increased susceptibility to MS. Although the Mendelian randomization approach used here largely avoids the possibility of confounding or reverse causation, the reliability of these findings may be limited by some of the assumptions made by the researchers during their analysis. Moreover, although these findings support a role for vitamin D in MS susceptibility, they provide no information about whether vitamin D modulates the course of MS after its onset, and they may not apply to people of non-European ancestry. Nevertheless, these findings provide a strong rationale for undertaking randomized controlled trials to investigate whether vitamin D supplementation can prevent the onset and/or progression of MS.

**Additional Information:**

This list of resources contains links that can be accessed when viewing the PDF on a device or via the online version of the article at http://dx.doi.org/10.1371/journal.pmed.1001866.

- The UK National Health Service Choices website provides information about multiple sclerosis, including personal stories, and information about vitamin D and how to get vitamin D from sunlight; A “Behind the Headlines” article examines health claims made for vitamin D
- The US National Institute of Neurological Disorders and Stroke provides information about multiple sclerosis and links to MS societies
- The National Multiple Sclerosis Society, a US not-for-profit organization, provides information about all aspects of MS, including information about vitamin D and MS and personal stories about MS
- The Multiple Sclerosis Society, a UK not-for-profit organization, also provides information about MS for patients, carers, and professionals in several languages, including news about the latest research, personal blogs, and forums
- The US Office of Dietary Supplements provides information about vitamin D (in English and Spanish)
- MedlinePlus has links to further information about multiple sclerosis and vitamin D (in English and Spanish)
- Wikipedia has a page on Mendelian randomization (note: Wikipedia is a free online encyclopedia that anyone can edit; available in several languages)

## Introduction

Multiple sclerosis (MS) is the most common permanent neurological disorder affecting young adults [1]. It is a debilitating autoimmune condition that presents early in life, with a mean age of onset of 28–31 y. Epidemiological studies have indicated that the prevalence of MS varies geographically, such that regions of higher latitude and with decreased levels of sunlight exposure have a higher prevalence of MS [2,3]. Since the circulating level of vitamin D, as measured by the level of 25-hydroxyvitamin D (25OHD, the clinical determinant of vitamin D status), is partially derived from sunlight exposure, it has been suggested that 25OHD deficiency may be the causal risk factor mediating this latitudinal gradient [4]. Further evidence to support the vitamin D hypothesis arose from the Nurses’ Health Study, which reported a protective effect on MS for women who had high levels of daily vitamin D intake [5]. Lower vitamin D level has also been associated with higher rates of MS relapse [6] and higher MS-specific disease activity and disability [7]. Vitamin D has important effects upon the immune system, and its immune-modulating effects have been observed in multiple cell-culture experiments [8], providing possible biological mechanisms whereby vitamin D may influence MS risk.

To date, there has been one published meta-analysis investigating the effect of vitamin D supplementation on MS relapse; the meta-analysis included five randomized controlled trials (RCTs) with a total of 254 participants [9]. The authors reported that the effect of high-dose vitamin D treatment on MS relapse was inconclusive (odds ratio [OR] = 0.98, 95% CI: 0.45–2.16) and that these trials had important methodological limitations, such as small sample size and short duration of vitamin D treatment. In contrast, two non-blinded trials demonstrated improved clinical outcomes with vitamin D therapy; however, disease activity or MRI changes were not the primary outcome of these trials [10,11]. Importantly, all of these trials test whether vitamin D can *treat* MS, but provide no insight into whether vitamin D can *prevent* MS.

Consequently, clinical practice guidelines for the treatment of MS [12] do not include vitamin D therapy. This is at least partially attributable to the possibility of confounding in the above observational studies. Additionally, observational studies are prone to reverse causation, where, for example, individuals with MS may spend less time outdoors and may as a result have lower circulating 25OHD levels. However, if decreased 25OHD levels are causally associated with MS, this could have important implications since vitamin D insufficiency, defined as 25OHD level < 50 nmol/l, is common and increasing in prevalence. This was observed in the National Health and Nutrition Examination Survey (NHANES): in 2005, 41.6% of adult Americans were found to be vitamin D insufficient, and mean 25OHD level decreased from 75 nmol/l in 1988 to 50 nmol/l in 2006 [13,14].

In the absence of high-quality RCT data, the principles of Mendelian randomization (MR) can be applied to strengthen or refute the causality of biomarkers in disease etiology [15]. MR analysis uses genetic associations to test the effects of biomarkers, such as 25OHD, on the risk of disease. This approach, which is conceptually similar to an RCT, is based on the principle that genetic variants are randomly allocated at meiosis, and consequently these genetic variants are independent of many factors that bias observational studies, such as confounding and reverse causation. MR methods have been used previously to investigate the role of high-density lipoprotein [16] and C-reactive protein [17] in predisposition to cardiovascular disease, and have provided strong evidence that PCSK9 inhibition prevents cardiovascular disease [18]. MR methods may be of particular relevance for understanding the etiology of MS since the date of disease onset is often poorly recognized clinically and MR studies assess the effect of lifetime exposures.

Here we adopted an MR design to clarify whether 25OHD level lies in the causal pathway for MS susceptibility. In order to assess whether a reduced level of 25OHD is associated with an increased risk of MS, we selected genome-wide significant single nucleotide polymorphisms (SNPs) as identified by SUNLIGHT (Study of Underlying Genetic Determinants of Vitamin D and Highly Related Traits), the largest genome-wide association study (GWAS) published to date for 25OHD level. Next, we estimated the effect of each of these SNPs upon 25OHD level in the Canadian Multicentre Osteoporosis Study (CaMos) and tested their validity as instrumental variables for MR analyses. Finally, we applied the principles of MR to investigate the association of a lifetime of genetically lowered 25OHD level with MS risk using data from the International Multiple Sclerosis Genetics Consortium (IMSGC).

## Methods

### SNP Selection and Data Sources

Genetic variants associated with 25OHD level at a genome-wide significant level (*p* < 5 × 10^−8^) were obtained from SUNLIGHT [19], a GWAS consisting of 33,996 individuals of European descent from 15 cohorts. 25OHD level in this study was measured by radioimmunoassay, chemiluminescent assay, ELISA, or mass spectrometry. Given that different cohorts used different methods to measure 25OHD level, results were combined across cohorts in SUNLIGHT using Z-score-weighted meta-analysis.

CaMos was used to estimate the effect of each genome-wide significant SNP on 25OHD level, since the effect of each SNP upon 25OHD level could not be used from SUNLIGHT, because of the Z-score meta-analytic approach employed [20]. CaMos is a large population-based cohort and was among the largest included in the replication phase of SUNLIGHT. It includes 2,347 individuals who were genotyped using TaqMan genotyping at the same genome-wide significant vitamin D loci found in SUNLIGHT.

To obtain precise estimates for the association of 25OHD with MS, we tested the effect of each genome-wide significant SNP for vitamin D level in the IMSGC Immunochip study, the largest international genetic study of MS, involving 14,498 MS cases and 24,091 healthy controls [21]. All participants were of European ancestry and were genotyped using the Immunochip array, which is a custom array designed to interrogate SNPs with potential immune system effects. Cases were defined as individuals diagnosed by a neurologist according to recognized diagnostic criteria dependent on laboratory and clinical information [22–24]. When data were not available for a specific SNP in the IMSGC Immunochip study, we used data from the second largest MS genetic study, the IMSGC study and IMSGC/Wellcome Trust Case Control Consortium 2 (IMSGC/WTCCC2) study, which included 9,772 cases and 6,332 controls taken from the IMSGC/WTCCC2 common control set [25].

### SNP Validation and Effect Sizes

#### Linkage disequilibrium assessment

One requirement of MR studies is that the selected SNPs must not be in linkage disequilibrium (LD) since if a selected SNP is highly correlated with other risk factor loci, this may result in confounding [15]. In order to verify that the SNPs in this study met this requirement, we measured LD between all selected SNPs using CEU samples from the 1000 Genomes Project (*n* = 94) [26].

#### Pleiotropy assessment

MR analyses assume that the chosen SNPs do not exert pleiotropic effects on the outcome (in this case, MS) by operating through biological pathways independent of the exposure (in this case, 25OHD level). However, in MR, a SNP may influence the outcome via other factors if the SNP acts upon the other factors *through* the exposure itself [27]. Previous work has assessed possible pleiotropic actions of the 25OHD-related SNPs used in our analysis by investigating the association between 25OHD-related SNPs and clinical traits in the 1958 British Birth Cohort, which included 6,877 participants of European descent [28]. In this cohort, no associations were found between these SNPs and relevant potential pleiotropic pathways, such as sun exposure, time outside, physical activity, oily fish consumption, smoking, alcohol consumption, body mass index (BMI), abdominal obesity, or social class (*p >* 0.05 for all) [28]. However, we note that some of these factors, such as sun exposure, time outside, BMI, and abdominal obesity, could act at least partially through the vitamin D pathway. Furthermore, SNPs associated with 25OHD level did not associate with other biomarkers (including C-reactive protein, IgE level, von Willebrand factor, tissue plasminogen activator, D-dimer, fibrinogen, triglyceride level, high-density lipoprotein, low-density lipoprotein, total cholesterol, forced expiratory volume, diastolic blood pressure, IGF-1, and HbA1c), and no interactions were observed between the SNPs, biomarkers, and 25OHD level. Additional details are provided in S1 Table.

To further explore sources of pleiotropy, we also conducted a systematic literature search of gene name, gene mutation, and protein name to examine the published literature for possible pleiotropic mechanisms for any of our selected SNPs for effects on MS and autoimmunity using PubMed. Details of this method are described in S1 Methods.

#### Population stratification assessment

The 1958 British Birth Cohort has previously assessed the potential for population stratification of the 25OHD-associated SNPs, which is a potential source of bias in MR studies since differences in minor allele frequencies between populations may cause the SNP to be associated with both the ancestry and the outcome [28]. In the 1958 British Birth Cohort, each SNP was tested for association with geographic region, which was dichotomized as south and middle UK (southeast England, southwest England, greater London, East Anglia, Midlands, and Wales) versus northern England (north England, northwest England, Yorkshire, and the Humber) and Scotland. We then further assessed potential population stratification of each SNP by testing its association with self-declared ethnicity in the CaMos cohort and tested the association of each SNP with non-European status, defined as exclusion from the European cluster in principal component analysis (PCA).

#### Effect size estimates of SUNLIGHT SNPs upon 25OHD level

To obtain the effect of each SNP upon 25OHD level, as required for MR analysis, we tested the additive effect of each minor allele on natural-log-transformed 25OHD level in CaMos, while controlling for sex, age, age squared, BMI, and season of 25OHD measurement (using categorical variables for summer [July–September], autumn [October–December], winter [January–March], and spring [April–June]) [19]. Ethnicity was checked by self-report and verified using PCA. To prevent population stratification from confounding our results, individuals who did not cluster with other Europeans were excluded from this analysis and were not used to measure the effect of each SNP upon 25OHD. The number of 25OHD-decreasing alleles was calculated for each participant in the CaMos cohort. This allele count was tested for an association with natural-log-transformed 25OHD level using linear regression, which had been residualized for the above covariates, and the F-statistic for the allele score was reported. The multiply adjusted natural-log-transformed 25OHD level was then assessed for each category of allele count, and a non-parametric trend test across these allele counts was computed.

### Association of SUNLIGHT SNPs with Multiple Sclerosis Susceptibility

In order to increase study power and obtain the most precise estimate of the association of 25OHD-associated SNPs with risk of MS, we used summary-level data from the IMSGC Immunochip study, if available (as described above). However, the IMSGC Immunochip genotyping array used was not genome-wide, so not all SNPs were captured in this experiment. If a SNP was not included in the IMSGC Immunochip study, then summary statistics from the second largest genotyped cohort, the IMSGC/WTCCC2 cohort, were selected. In the event that a SNP was not genotyped in either cohort, summary statistics for a perfect proxy SNP, defined as a surrogate SNP with perfect LD (*r* ^2^ = 1.0) to the SNP interest, were selected. LD for proxy SNPs was calculated using CEU samples from the 1000 Genomes Project (*n* = 94) since the IMSGC samples are of the same ancestry [26]. We then assessed whether each SNP was associated with the risk of MS, applying a Bonferroni correction, where statistical significance was declared at *p* ≤ 0.05/*n* where *n* is the number of SNPs associated with 25OHD level from SUNLIGHT.

### Mendelian Randomization Estimates

We conducted our MR analysis by assessing the effects of the SNPs upon risk of MS, weighting the effect of each SNP by the magnitude of its effect upon 25OHD level. In this study design, which has been described previously [29–31], the independent SNPs evaluate the association of exposure to genetically lowered 25OHD with MS risk. The individual estimates of the effect were then pooled using statistically efficient estimators formally analogous to those of inverse-variance-weighted meta-analysis [32]. We carried out a meta-analysis of estimates obtained from individual 25OHD-decreasing alleles using both fixed-effects and random-effects models to obtain pooled estimates for the combined effect of the 25OHD SNPs on MS.

Specifically, let *x* and *y* denote the centered and scaled natural-log 25OHD and log-odds MS traits, respectively, and suppose these are related by the linear structural equation: *y* = α*x +* η. Here, η is a stochastic error term, and in general *x* and η are correlated because of confounding. The parameter α quantifies the causal effect of *x* on *y*, and is thus the parameter we seek to estimate. Let *u*_*i*_ denote the allele dosage variable of the *i* ^th^ genetic variant. Let *γ*_*i*_ and β_*i*_ denote effect size estimates (derived from GWAS data) of *u*_*i*_ on the exposure *x* (change in natural-log 25OHD level) and outcome *y* (change in log odds of MS), respectively, and let *s*(β_*i*_) denote the standard error of β_*i*_. Then the MR estimate associated with the *i* ^th^ genetic variant is α_*i*_ = β_*i*_ */γ*_*i*_, and the variance of this estimate is *v*_*i*_ = (*s*[β_i_])/*γ*_*i*_)^2^. Define the precision of the *i* ^th^ MR estimate of α by *w*_*i*_ = *1*/*v*_*i*_. The inverse-variance-weighted fixed-effects estimate is then

$$\alpha_{\text{fixed}}=\frac{\sum_{i=1}^{n}w_{i}\alpha_{i}}{\sum_{i=1}^{n}w_{i}}$$ (1)

and the standard error *s*(α_fixed_) of this estimate is given by

s(αfixed)=(∑i=1wi)−12 (2)

We observe that α_fixed_ may also be interpreted as the regression coefficient resulting from the generalized linear regression of the outcome effect sizes β_*i*_ on the exposure effect sizes *γ*_*i*_ assuming heteroskedastic errors; in this regression, the *i* ^th^ error term has a variance equal to *s*(β_*i*_)^2^, and the offset coefficient in the regression is constrained to be zero.

The random-effects estimate α_random_ and its standard error *s*(α_random_) were also constructed from the individual estimates using standard methods [33] in which the weights are adjusted to account for the intrinsic variability (or heterogeneity) in the effect size. Heterogeneity may be quantified with the parameter *I* ^2^, which reports the fraction of the total variance in the meta-analytic estimate that is due to intrinsic variability in the effect size, as distinct from variability arising from measurement error [34]. The random-effects estimate α_random_ and its standard error *s*(α_random_) are given by equations analogous to those for α_fixed_ and *s*(α_fixed_), in which the weights assigned to individual estimates are adjusted to take into account heterogeneity in the effect size.

For all MR meta-analyses, we report estimates using both fixed-effects and random-effects models. The effect size for each meta-analysis is reported in the main results as the effect of a one-standard-deviation (1-SD) change in natural-log-transformed 25OHD level, since this metric is more interpretable than an arbitrary difference. This measure is given by exp(α_fixed_) for the fixed-effects model and by exp(α_random_) for the random-effects model. We also report the *I* ^2^ as an assessment of heterogeneity.

In order to provide a better clinical interpretation of a 1-SD change in natural-log-transformed 25OHD level, we selected three different clinically relevant 25OHD thresholds for vitamin D status (<25 nmol/l for vitamin D deficiency, <50 nmol/l for vitamin D insufficiency, and >75 nmol/l for vitamin D sufficiency) [35]. These thresholds were converted to the natural log scale because the magnitude of a 1-SD change is not constant on the untransformed scale. For each of these natural-log-transformed 25OHD levels, we then calculated a 1-SD increase in natural-log-transformed 25OHD. To obtain 25OHD levels that correspond to circulating levels in units of nanomoles/liter, we then back-transformed these values.

### Sensitivity Analyses

MR estimates were recalculated after exclusion of SNPs potentially influenced by pleiotropy or population stratification. Since SNPs associated with 25OHD level in SUNLIGHT influence either 25OHD synthesis or 25OHD metabolism [28], we elected to perform a stratified MR analysis where SNPs involved in either 25OHD synthesis or metabolism were analyzed separately.

## Results

### SNP Selection and Validation

#### SNP selection

**Fig 1:**
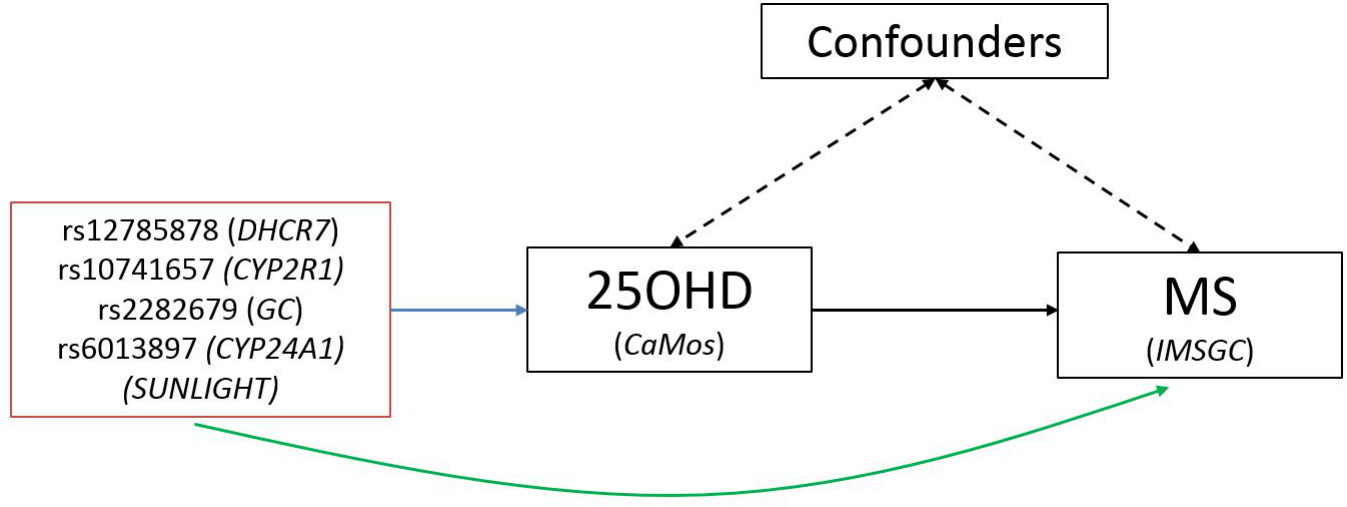
Schematic representation of Mendelian randomization analysis. The leftmost box lists SNPs that were genome-wide significant for 25OHD level in SUNLIGHT (*n* = 33,996). The blue arrow represents the effect of SNPs on multiply adjusted natural-log-transformed 25OHD level using data from CaMos (*n* = 2,347). The green arrow represents the causal association of decreased 25OHD level with the risk of MS using data from the largest genetic association study to date for MS (the IMSGC Immunochip study, up to 14,498 cases and 24,091 healthy controls).

**Fig 2:**
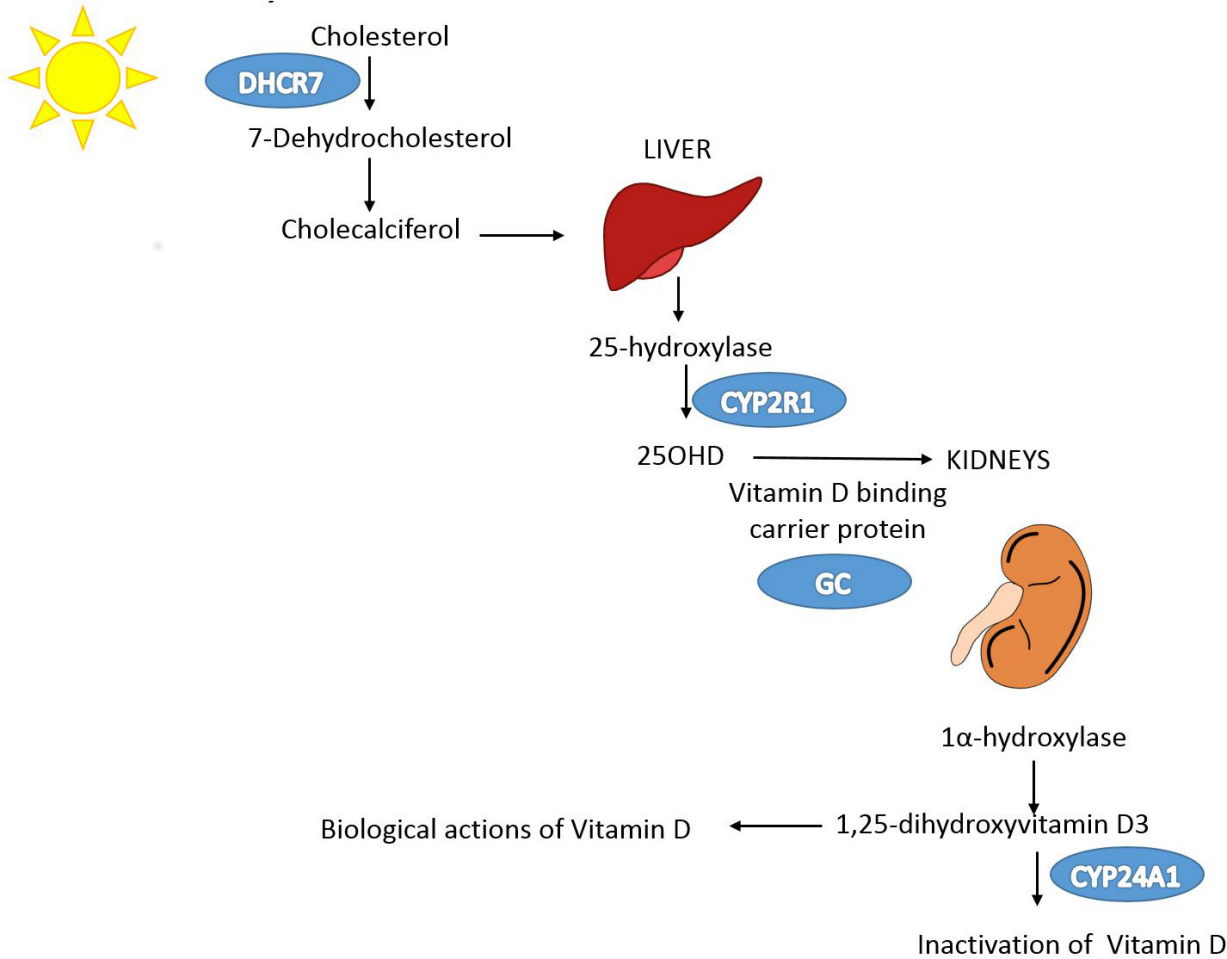
Vitamin D pathway. In blue are the genes containing, or in proximity to, SNPs that were genome-wide significant for 25OHD level in SUNLIGHT (*n* = 33,996). The *p*-values for the association with 25OHD level were 1.9 × 10^−109^ for *GC*, 2.1 × 10^−27^ for *DHCR7*, 3.3 × 10^−20^ for *CYP2R1*, and 6.0 × 10^−10^ for *CYP24A1*. Note that each gene plays an independent role in modulating the level of 25OHD. Kidney and liver images credit: https://openclipart.org/.

A schematic representation of the MR study design is presented in Fig 1. SUNLIGHT identified four SNPs as genome-wide significant for 25OHD level [19]. These included rs2282679 in *GC* (association with 25OHD: *p* = 1.9 × 10^−109^), rs12785878 near *DHCR7* (*p* = 2.1 × 10^−27^), rs10741657 near *CYP2R1* (*p* = 3.3 × 10^−20^), and rs6013897 in *CYP24A1* (*p* = 6.0 × 10^−10^). We selected these SNPs for our MR study since all are strongly associated with 25OHD level and map to genes implicated in the modulation of 25OHD level through distinct mechanisms [36]. Specifically, *GC* encodes the vitamin D binding protein (DBP), a group-specific component of serum globulin. DBP acts as the principal protein carrier for 25OHD, transporting 80%–90% of 25OHD to target organs [37–39]. The *DHCR7* gene product is known to convert 7-dehydrocholesterol to cholesterol, providing a substrate for vitamin D production. CYP2R1 is a regulator of 25OHD synthesis through 25-hydroxylation of vitamin D in the liver, the first activation step [40], and, lastly, CYP24A1 inactivates 1α25(OH)_2_D, rendering inactive the active form of vitamin D (Fig 2). Therefore, all SNPs used in this study map near genes strongly implicated in vitamin D synthesis, transport, or metabolism. Notably, all four SNPs lie in intergenic or intronic regions, and presently the exact effect of each SNP on these enzymes is unknown. Nevertheless, all SNPs reside near genes strongly implicated in vitamin D synthesis or metabolism [36].

#### Linkage disequilibrium and pleiotropy assessment

There was no evidence of LD between any of the four 25OHD SNPs (all pairwise *r* ^2^ ≤ 0.01) in the 1000 Genomes Project CEU samples. We note that only two of the SNPs, rs10741657 and rs12785878, were located on the same chromosome, which greatly decreases the risk of confounding by LD. As described above, none of the four 25OHD SNPs was associated with relevant pleiotropic pathways in the 1958 British Birth Cohort.

Undertaking a literature review for possible pleiotropic pathways, we found no evidence for pleiotropic mechanisms for the vitamin D metabolism SNPs, rs10741657 (*CYP2R1*) and rs6013897 (*CYP24A1*). For rs2282679 (*GC*), we found that its encoded protein, DBP, has been associated with macrophage activation and may modulate T cell response to vitamin D [41]. Elevated DBP levels are found in the cerebrospinal fluid of patients with Alzheimer disease [42] and MS [43], and have been linked to the progression of MS in rats [44]. It has been argued that DBP can act independently of vitamin D to produce clinical phenotypes; therefore, we undertook sensitivity analyses excluding rs2282679 (*GC*) in our MR analyses. Genetic variation in *DHCR7* appears to cause Smith-Lemli-Opitz syndrome, a clinical phenotype relating to cholesterol deficiency. Given that a recent study suggested an interdependence of cholesterol and vitamin D pathways in the etiology of MS [45], we queried the association of rs12785878 in the largest publically available GWAS results for lipids, those of the Global Lipids Genetics Consortium [46], and found that this SNP was associated with a minimum *p*-value of 0.043 across all lipid traits, suggesting that the SNP is not strongly associated with cholesterol.

#### Population stratification assessment

Previous reports from the 1958 British Birth Cohort demonstrated that rs12785878 (*DHCR7*) was associated with geographic region [28]. Since rs12785878 is unevenly distributed across geography and the prevalence of MS varies by geographic location, a potential surrogate for local ancestry [4], we tested whether this SNP was associated with non-European status in CaMos using PCA. The SNP rs12785878 was strongly associated with non-European status in the CaMos cohort (*p* = 2.7 × 10^−13^). No other SNP showed any evidence of correlation with non-European status (*p >* 0.5 for all other SNPs). Given this possible relationship with population stratification, we undertook MR sensitivity analyses excluding the rs12785878 (*DHCR7*) variant.

### Association of SUNLIGHT SNPs with 25OHD Level

**Fig 3:**
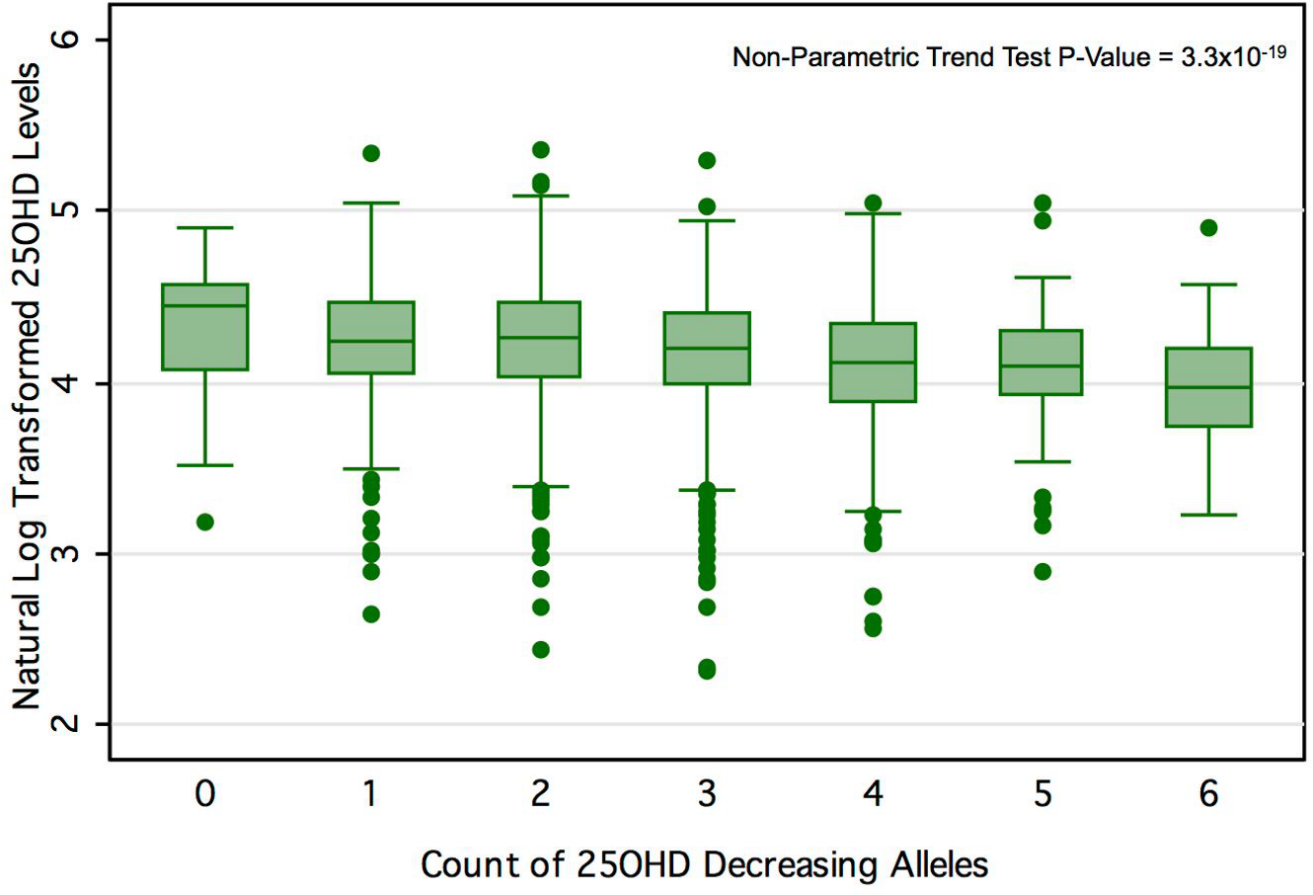
25OHD level by number of 25OHD-decreasing alleles in the CaMos cohort. Here we show the box-plot of natural-log-transformed 25OHD by the count of 25OHD-decreasing alleles in the CaMos population. A count of zero represents individuals with no 25OHD-decreasing alleles (or homozygous at each loci for the 25OHD-increasing allele), and a count of six represents an individual with six 25OHD-decreasing alleles. No individuals with a count of seven or more 25OHD-decreasing alleles were observed in this cohort. The center line and error bars represent the mean level of natural-log-transformed 25OHD and its 95% CI for each respective allele count. Note a negative trend between allele count and mean natural-log-transformed 25OHD.

Table 1 displays the four SNPs that achieved genome-wide significance for 25OHD level in SUNLIGHT and describes their association with 25OHD [19]. Each of these SNPs explained an important proportion of the population-level variance in 25OHD level, as reflected by the F-statistic. The count of 25OHD-decreasing alleles across these four SNPs was strongly associated lower 25OHD level in the CaMos population, residualized for age, season, sex, and BMI (F-statistic = 49.7, *r* ^2^ = 2.44%, *p* for allelic score = 2.4 × 10^−12^). Fig 3 shows the mean 25OHD levels for individuals with increasing counts of 25OHD-decreasing alleles (non-parametric trend test, *p* = 3.3 × 10^−19^).

**Table 1:** Characteristics of SNPs used as instrumental variables.

Locus	Chromosome	25OHD-Associated SNP	25OHD-Decreasing Allele	Allele Frequency	Vitamin D Results			MS Results		
					Effect on 25OHD^a^	*p*-Value for Association with 25OHD^b^	F-Statistic for 25OHD^c^	OR (95% CI) for MS	*p*-Value for Association with MS	Study
*CYP2R1*	11	rs10741657	C	0.62	−0.052	3.3 × 10^−20^	18.78	1.05 (1.02–1.09)	3.9 × 10^−3^	IMSGC Immunochip [21]
*DHCR7*	11	rs12785878	G	0.27	−0.056	2.1 × 10^−27^	18.29	1.11 (1.07–1.15)^d^	8.7 × 10^−9^	IMSGC Immunochip [21]
*GC*	4	rs2282679	C	0.30	−0.047	1.9 × 10^−109^	13.38	1.04 (1–1.08)	6.2 × 10^−2^	IMSGC/ WTCCC2 [25]
*CYP24A1*	20	rs6013897	A	0.19	−0.027	6.0 × 10^−10^	3.13	1.07 (1.03–1.11)^e^	1.7 × 10^−3^	IMSGC/ WTCCC2 [25]

^a^Effect on multiply adjusted natural-log-transformed 25OHD level in the CaMos cohort.

^b^ *p*-Values derived from SUNLIGHT.

^c^F-statistic derived from multiply adjusted natural-log-transformed 25OHD level in the CaMos cohort.

^d^SNP rs12785878 was not available for MS data. Therefore SNP rs4944958 was used as a proxy (*r* ^2^ between these two SNPs = 1.0).

^e^SNP rs6013897 was not available for MS data. Therefore SNP rs17217119 was used as a proxy (*r* ^2^ between these two SNPs = 1.0).

### Association of SUNLIGHT SNPs with Multiple Sclerosis Susceptibility

Summary statistics for two of the four 25OHD-associated SNPs (rs10741657 at *CYP2R1* and rs12785878 at *DHCR7*) and their association with MS were taken from the IMSGC Immunochip study (Table 1). rs12785878 at *DHCR7* was not directly genotyped in the IMSGC Immunochip study; however, a perfect proxy for rs12785878, rs4944958, was used (*r* ^2^ = 1.0 between rs12785878 and rs4944958 in the 1000 Genomes Project CEU samples). Summary statistics for the remaining two SNPs (rs6013897 at *CYP24A1* and rs2282679 at *GC*) were taken from the second largest MS genetic association study, the IMSGC/WTCCC2 study. SNP rs6013897 at *CYP24A1* was not present in the IMSGC/WTCCC2 dataset, and therefore a perfect proxy SNP for rs6013897, rs17217119, was used (*r* ^2^ = 1.0 between rs17217119 and rs6013897 from the 1000 Genomes Project CEU samples).

All four 25OHD-decreasing alleles were associated with an increased risk of MS (Table 1). rs12785878 (*DHCR7*) achieved genome-wide significance for MS risk, while two 25OHD-decreasing alleles (rs10741657 and rs6013897) were moderately associated with MS risk (*p* = 3.9 × 10^−3^ and *p* = 1.7 × 10^−3^, respectively). The 25OHD-decreasing allele rs2282679 (allele C) (*GC*) was not significantly associated with MS risk (*p* = 0.062) (Table 1). However, three of the 25OHD-associated SNPs (rs12785878, rs10741657, and rs6013897) remained associated with MS after a Bonferroni correction for the number of independent SNPs (*p* ≤ 0.05/4 = 0.0125).

### Mendelian Randomization Analysis for the Association of 25OHD with Multiple Sclerosis Risk

**Fig 4:**
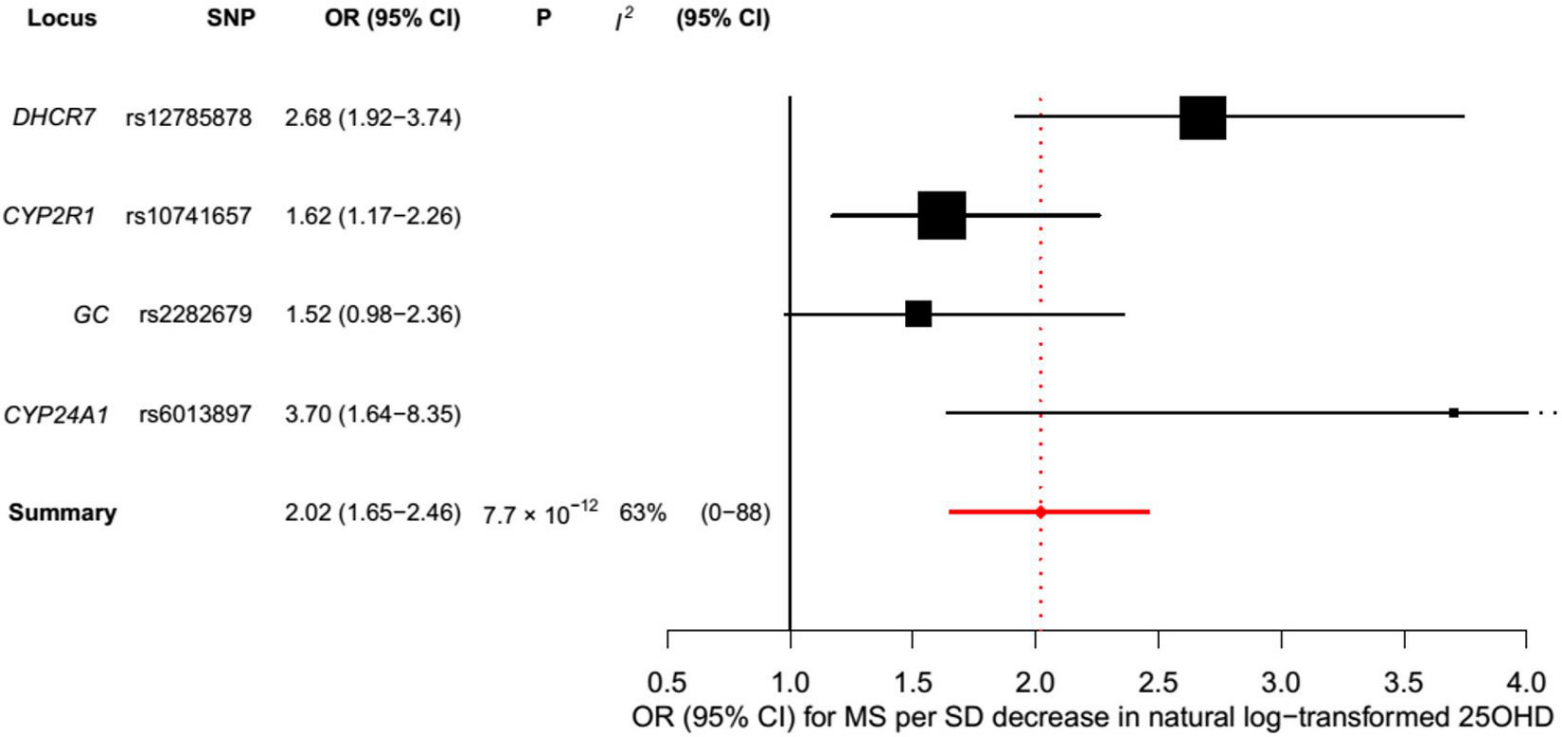
Mendelian randomization estimate of the association of 25OHD level with risk of multiple sclerosis. Estimates obtained using a fixed-effects model.

**Fig 5:**
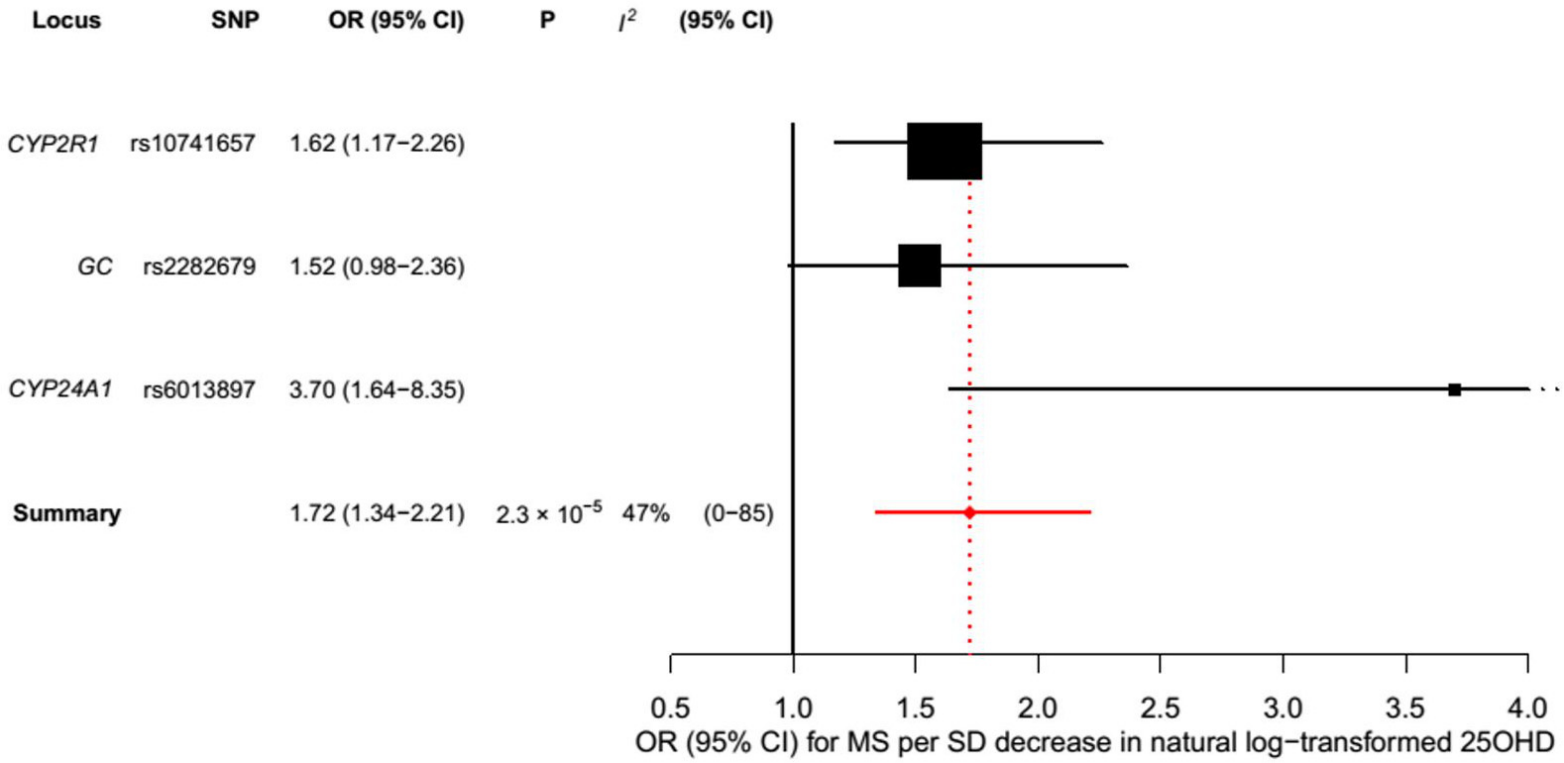
Mendelian randomization estimate of the association of 25OHD level with risk of multiple sclerosis excluding the *DHCR7* locus. Estimates obtained using a fixed-effects model.

In order to estimate the association of genetically lowered 25OHD with MS, we used a fixed-effects model in which all four 25OHD-decreasing alleles of the MR set were included. We observed that each 1-SD decrease in natural-log-transformed 25OHD level was associated with an increased risk of MS (OR = 2.02, 95% CI: 1.65–2.46, *p* = 7.72 × 10^−12^) (Table 2; Fig 4). Given that the *I* ^2^ estimate of heterogeneity was somewhat increased (*I* ^2^ = 63%, 95% CI: 0%–88%), we also undertook random-effects meta-analysis, which generated similar findings (OR = 2.07, 95% CI: 1.45–2.96, *p* = 5.74 × 10^−5^) (Table 2; S1 Fig). We note that since our model included only four SNPs, the 95% CIs of the *I* ^2^ statistic are wide, and consequently heterogeneity cannot be accurately measured using this parameter. In addition, to address the potential effects of population stratification and pleiotropy, we undertook a sensitivity analysis excluding the rs12785878 SNP (*DHCR7*). Despite removal of this variant, we observed a clear association of genetically lowered 25OHD level with the risk of MS (OR = 1.72, 95% CI: 1.34–2.21, *p* = 2.28 × 10^−5^; *I* ^2^ = 47%, 95% CI: 0%–85%) (Table 3; Fig 5), which remained significant using a random-effects meta-analysis (OR = 1.82, 95% CI: 1.24–2.67, *p* = 2.13 × 10^−3^; *I* ^2^ = 47%, 95% CI: 0%–85%) (Table 3; S2 Fig). Removal of the rs2282679 SNP (*GC*), which may possibly be influenced by pleiotropy, did not influence the MR results using a fixed-effects or random-effects model (OR = 2.17, 95% CI: 1.73–2.72, *p* = 1.7 × 10^−11^; *I* ^2^ = 67%, 95% CI: 0%–91%; and OR = 2.32, 95% CI: 1.49–3.61, *p* = 1.8 × 10^−4^; *I* ^2^ = 67%, 95% CI: 0%–90%, respectively) (S3 and S4 Figs). To further assess the effect of the independent vitamin D pathways on the risk of MS, we analyzed SNPs near genes implicated in 25OHD synthesis (*DHCR7* and *CYP2R1*) and metabolism (*GC* and *CYP24A1*) separately and found that both strongly associated with increased risk of MS (Table 4; S5 and S6 Figs).

**Table 2:** Mendelian randomization estimate of the association of decreased 25OHD with the risk of multiple sclerosis.

Model	OR (95% CI)^a^	*p*-Value	*I* ^2^ (95% CI)
Fixed effects	2.02 (1.65–2.46)	7.72 × 10^−12^	63% (0%–88%)
Random effects	2.07 (1.45–2.96)	5.74 × 10^−5^	63% (0%–88%)

^a^OR is expressed as the odds of MS for a 1-SD decrease in natural-log-transformed 25OHD level.

**Table 3:** Mendelian randomization estimate of the association of decreased 25OHD with the risk of multiple sclerosis excluding the *DHCR7* locus.

Model	OR (95% CI)^a^	*p*-Value	*I* ^2^ (95% CI)
Fixed effects	1.72 (1.34–2.21)	2.28 × 10^−5^	47% (0%–85%)
Random effects	1.82 (1.24–2.67)	2.13 × 10^−3^	47% (0%–85%)

^a^OR is expressed as the odds of MS for a 1-SD decrease in natural-log-transformed 25OHD level.

**Table 4:** Mendelian randomization estimate of the association of decreased 25OHD with the risk of multiple sclerosis stratified by SNPs near genes involved in 25OHD synthesis versus metabolism using a fixed-effects model.

Model	OR (95% CI)^a^	*p*-Value
25OHD synthesis	2.08 (1.64–2.63)	1.1 × 10^−9^
25OHD metabolism	1.86 (1.26–2.74)	1.7 × 10^−3^

^a^OR is expressed as the odds of MS for a 1-SD decrease in natural-log-transformed 25OHD level. Note that the 95% CI for the *I* ^2^ cannot be properly estimated given that there are only two SNPs per model.

The clinical equivalences of a 1-SD increase in natural-log-transformed 25OHD for the thresholds for vitamin D deficiency (<25 nmol/l), vitamin D insufficiency (<50 nmol/l), and vitamin D sufficiency (>75 nmol/l) are shown in Table 5. We observed that for individuals at the threshold for vitamin D deficiency (25OHD = 25 nmol/l), an increase in 25OHD level to 36.9 nmol/l would be required to decrease the odds of MS by 50%, while for individuals at the thresholds for vitamin D insufficiency (25OHD = 50 nmol/l) and vitamin D sufficiency (25OHD = 75 nmol/l), an increase in 25OHD level to 73.7 nmol/l and 110.6 nmol/l, respectively, would similarly be required.

**Table 5:** Clinical equivalence of a 1-SD natural-log increase in 25OHD for various vitamin D thresholds.

Clinically Relevant 25OHD Threshold	25OHD Level Required to Decrease Odds of MS by 50%^a^
Vitamin D deficient (25 nmol/l)	36.86 nmol/l
Vitamin D insufficient (50 nmol/l)	73.72 nmol/l
Vitamin D sufficient (75 nmol/l)	110.6 nmol/l

^**a**^Expressed as the equivalent of a natural-log-transformed SD increase in 25OHD on the nanomoles/liter scale.

## Discussion

Using summary-level data for MS and 25OHD levels from large European populations, our study demonstrated that a genetic decrease in natural-log-transformed 25OHD by 1 SD was associated with a 2-fold increase in risk of MS, providing strong evidence in support of a causal role of vitamin D in MS susceptibility. These findings are consistent with evidence from observational studies that have demonstrated that low vitamin D levels influence risk of MS and also reflect findings from functional studies that have implicated vitamin D as an important regulator in the expression of MHC class II genes [47,48]. This evidence provides rationale to further investigate whether vitamin D supplementation may reduce MS susceptibility in those most at risk.

The identification of vitamin D as a causal susceptibility factor for MS may have important public health implications since vitamin D insufficiency is common [13,14], and vitamin D supplementation is both relatively safe and cost-effective [35]. The importance of these findings may be magnified in high-latitude countries, which have disproportionately higher rates of MS and also higher rates of vitamin D insufficiency.

A reasonable first step to understanding the role of vitamin D therapy in delaying the onset or severity of MS would be to treat vitamin D insufficiency in those most at risk of developing MS. MS is often preceded by clinically isolated syndrome, which is a first clinical episode compatible with MS, often accompanied by lesions on magnetic resonance imaging [44], thereby providing a therapeutic window and rationale for intervening with vitamin D supplementation. Ongoing RCTs are currently assessing vitamin D supplementation for the treatment and prevention of MS [49,50] and may therefore provide needed insights into the role of vitamin D supplementation.

An important difference between MR studies and RCTs is that MR studies describe the association of a *lifetime of exposure* to vitamin-D-lowering alleles in the general population, whereas RCTs provide insights from supplementation for shorter periods in individuals at risk. Thus, long-term RCTs may be needed to adequately assess the impact of vitamin D supplementation in the prevention or treatment of MS. Lastly, MR may be an ideal study design to understand risk factors for MS, given the long latency period between disease onset and diagnosis, since MR may permit the estimation of lifetime exposure to risk factors.

Our analysis has several strengths. First, by utilizing the random allocation of genetic variants, we were able to overcome potential confounding and reverse causation that may bias estimates from observational studies. Second, using data from the largest genetic consortia for 25OHD level (*n* = 33,996) and MS risk (up to 14,498 cases and 24,091 controls) has enabled us to more precisely test our study hypothesis than if we had used individual-level data from a small study. Previous work has shown that using estimates from meta-analytic data for uncorrelated genetic variants is similarly efficient to using individual-level data in MR studies [29]. Lastly, the findings from this study represent the association of a lifelong exposure to reduced vitamin D levels with MS in the general European population, and, in the absence of large-scale, long-term RCT data, our findings provide strong evidence in support of a causal role of low vitamin D levels in MS susceptibility.

Our study also has limitations. First, while we have provided evidence supporting a role for vitamin D in MS susceptibility, we cannot conclude that vitamin D plays a role in disease modulation after disease onset. While MR is able to overcome the limitations that may bias observational studies, the possibility of residual pleiotropy could bias estimates in this study. However, in this study the main findings remained robust in multiple sensitivity analyses testing the pleiotropy assumption, thereby decreasing the probability of bias due to pleiotropy. We also note that all four studied SNPs are located in or near 25OHD-associated genes and influence 25OHD levels through known and distinct mechanisms. Additionally, the point estimate for each 25OHD-decreasing allele, as well as the combined 25OHD synthesis and metabolism pathways, was independently associated with increased risk of MS. Therefore, it is unlikely that pleiotropy strongly biased our results. Like in most MR studies, we cannot directly assess whether canalization, which is defined as compensatory feedback interactions, may have influenced our results [15,51,52]. However, since canalization assumes that other physiological mechanisms may attenuate the effect of genetically reduced 25OHD levels, such feedback interactions would tend to bias results toward the null. In contrast, our study has generated results that are very distinct from the null.

MR analyses using *DHCR7*, *GC*, *CYP24A1*, and *CYP2R1* as instruments have been performed in the past [53–57]. We and others recently provided evidence from MR that low vitamin D levels do not increase insulin resistance [53] or the risk of type 2 diabetes [53,54] or coronary heart disease [53], but do increase the risk of type 1 diabetes [55] and possibly blood pressure [56]. Interestingly, MR has shown that 25OHD levels are directly influenced by BMI, and converse effects are likely to be small [57]. Thus, while observational associations between 25OHD and two autoimmune conditions—type 1 diabetes and now MS—have been supported by genetic evidence, associations with cardio-metabolic outcomes have not been supported thus far.

In conclusion, using data from the largest existing genetic consortia, we demonstrate that genetically lowered 25OHD level is associated with an increase in the risk of MS in people of European descent. These findings provide rationale for further investigating the potential therapeutic benefits of vitamin D supplementation in preventing the onset and progression of MS.

## Supporting Information

S1 Methods Details of our systematic PubMed search exploring possible sources of pleiotropy. (PDF)

S1 Fig MR estimate of the association of 25OHD level with risk of MS using a random-effects model. (PDF)

S2 Fig MR estimate of the association of 25OHD level with risk of MS excluding the *DHCR7* locus using a random-effects model. (PDF)

S3 Fig MR estimate of the association of 25OHD level with risk of MS excluding the *GC* locus using a fixed-effects model. (PDF)

S4 Fig MR estimate of the association of 25OHD level with risk of MS excluding the *GC* locus using a random-effects model. (PDF)

S5 Fig MR estimate of the association of 25OHD level with risk of MS for SNPs involved in 25OHD synthesis using a fixed-effects model. (PDF)

S6 Fig MR estimate of the association of 25OHD level with risk of MS for SNPs involved in 25OHD metabolism using a fixed-effects model. (PDF)

S1 Table Summary of previous work assessing the association between 25OHD SNPs and relevant biomarker pathways. (PDF)

## Abbreviations:

25OHD: 25-hydroxyvitamin D
BMI: body mass index
CaMos: Canadian Multicentre Osteoporosis Study
GWAS: genome-wide association study
IMSGC: International Multiple Sclerosis Genetics Consortium
LD: linkage disequilibrium
MR: Mendelian randomization
MS: multiple sclerosis
NHANES: National Health and Nutrition Examination Survey
OR: odds ratio
PCA: principal component analysis
RCT: randomized controlled trial
SD: standard deviation
SNP: single nucleotide polymorphism
SUNLIGHT: Study of Underlying Genetic Determinants of Vitamin D and Highly Related Traits
WTCCC2: Wellcome Trust Case Control Consortium 2
