# Correction: Vitamin D and Risk of Multiple Sclerosis: A Mendelian Randomization Study

**DOI:** 10.1371/journal.pmed.1001981

**Published:** 2016-03-02

**Authors:** Lauren E. Mokry, Stephanie Ross, Omar S. Ahmad, Vincenzo Forgetta, George Davey Smith, David Goltzman, Aaron Leong, Celia M. T. Greenwood, George Thanassoulis, J. Brent Richards

Dr. David Goltzman is not included in the author byline. He should be listed as the sixth author and affiliated with Department of Medicine, McGill University, Montreal, Quebec, Canada. The contributions of this author are as follows: Contributed to the application of the methods and the interpretation of the findings.

The correct citation is: Mokry LE, Ross S, Ahmad OS, Forgetta V, Smith GD, Goltzman D, et al. (2015) Vitamin D and Risk of Multiple Sclerosis: A Mendelian Randomization Study. PLoS Med 12(8): e1001866. doi:10.1371/journal.pmed.1001866

In addition, Fig 2 is incorrect. The authors have provided a corrected version here. The direction of one arrow has been changed, and the label ‘UVB’ has been added.

**Fig 2:**
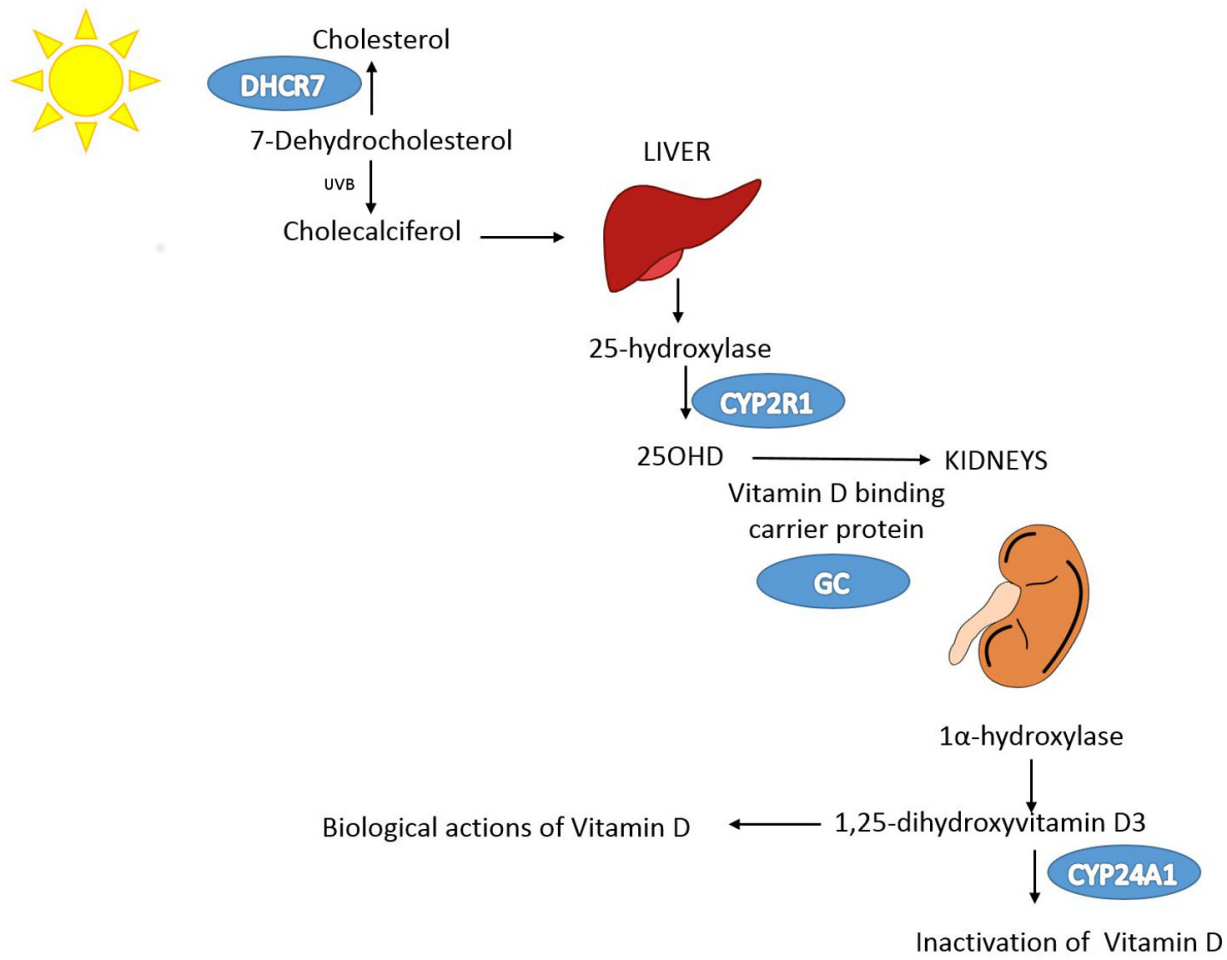
Vitamin D pathway. In blue are the genes containing, or in proximity to, SNPs that were genome-wide significant for 25OHD level in SUNLIGHT (n = 33,996). The p-values for the association with 25OHD level were 1.9 × 10−109 for GC, 2.1 × 10−27 for DHCR7, 3.3 × 10−20 for CYP2R1, and 6.0 × 10−10 for CYP24A1. Note that each gene plays an independent role in modulating the level of 25OHD. Kidney and liver images credit: https://openclipart.org/.
