# Supplementary material for: Vitamin D and Risk of Multiple Sclerosis: A Mendelian Randomization Study
Source: PLoS Med. 2015 Aug 25;12(8):e1001866. doi: 10.1371/journal.pmed.1001866 (PMC4549308; doi:10.1371/journal.pmed.1001866)
Supplement: S1 Methods — (PDF) [file pmed.1001866.s001.pdf]

Vitamin D and risk of Multiple Sclerosis: a Mendelian Randomization Study

Lauren E Mokry, Stephanie Ross, Omar S. Ahmad, Vincenzo Forgetta, George Davey-Smith, Aaron Leong, Celia MT Greenwood, George Thanassoulis, J. Brent Richards

SUPPLEMENTARY METHODS

PubMed Search

The following terms were searched on the PubMed database to investigate possible pleiotropic mechanisms of our chosen SNPs corresponding to gene name, gene mutations, encoded protein, encoded protein with MS and encoded protein with autoimmunity.

For rs2282679: “*GC”, “GC gene”, “GC gene mutations”, “vitamin D binding protein”, “vitamin D binding protein multiple sclerosis”, “vitamin D binding protein autoimmunity”,*

The search term GC uncovered 69152 results, most of which were not relevant to genetics, therefore the search term “GC gene” was used instead to refine search results.

For rs12785878: “*DHCR7”, “DHCR7 mutations”, “7-dehydrocholesterol reductase”, “7-dehydrocholesterol reductase multiple sclerosis”, 7-dehydrocholesterol reductase autoimmunity”*

For rs6013897: “*CYP24A1”, “CYP24A1 mutations”, “1,25-dihydroxyvitamin D3 24-hydroxylase”, “1,25-dihydroxyvitamin D3 24-hydroxylase multiple sclerosis” “1,25-dihydroxyvitamin D3 24-hydroxylase autoimmunity”*

For rs10741657: “*CYP2R1”, “CYP2R1 mutations”, “vitamin-D hydroxylase”, “vitamin-D hydroxylase multiple sclerosis”, “vitamin-D hydroxylase autoimmunity”.*

Abstracts were selected for further review if they made reference to the search term and a pathway distinct from vitamin D or vitamin D insufficiency/ deficiency on MS or autoimmunity. Only studies in mammals were considered. Vitamin-D related genes have often been associated with other clinical phenotypes such as colorectal cancer and inflammatory airway conditions however these were considered unrelated to MS and autoimmunity. Findings are reported in the Results section.
