# Supplementary material for: Vitamin D and Risk of Multiple Sclerosis: A Mendelian Randomization Study
Source: PLoS Med. 2015 Aug 25;12(8):e1001866. doi: 10.1371/journal.pmed.1001866 (PMC4549308; doi:10.1371/journal.pmed.1001866)

Figure S1: Mendelian Randomization Estimate of the Association of 25OHD Levels with Risk of MS Using a Random Effects Model

Locus	SNP	OR (95% CI)	P	I^2	(95% CI)
DHCR7	rs12785878	2.68 (1.92−3.74)			
CYP2R1	rs10741657	1.62 (1.17−2.26)			
GC	rs2282679	1.52 (0.98−2.36)			
CYP24A1	rs6013897	3.70 (1.64−8.35)			
**Summary**		2.07 (1.45−2.96)	5.7×10^{-5}	63%	(0−88)

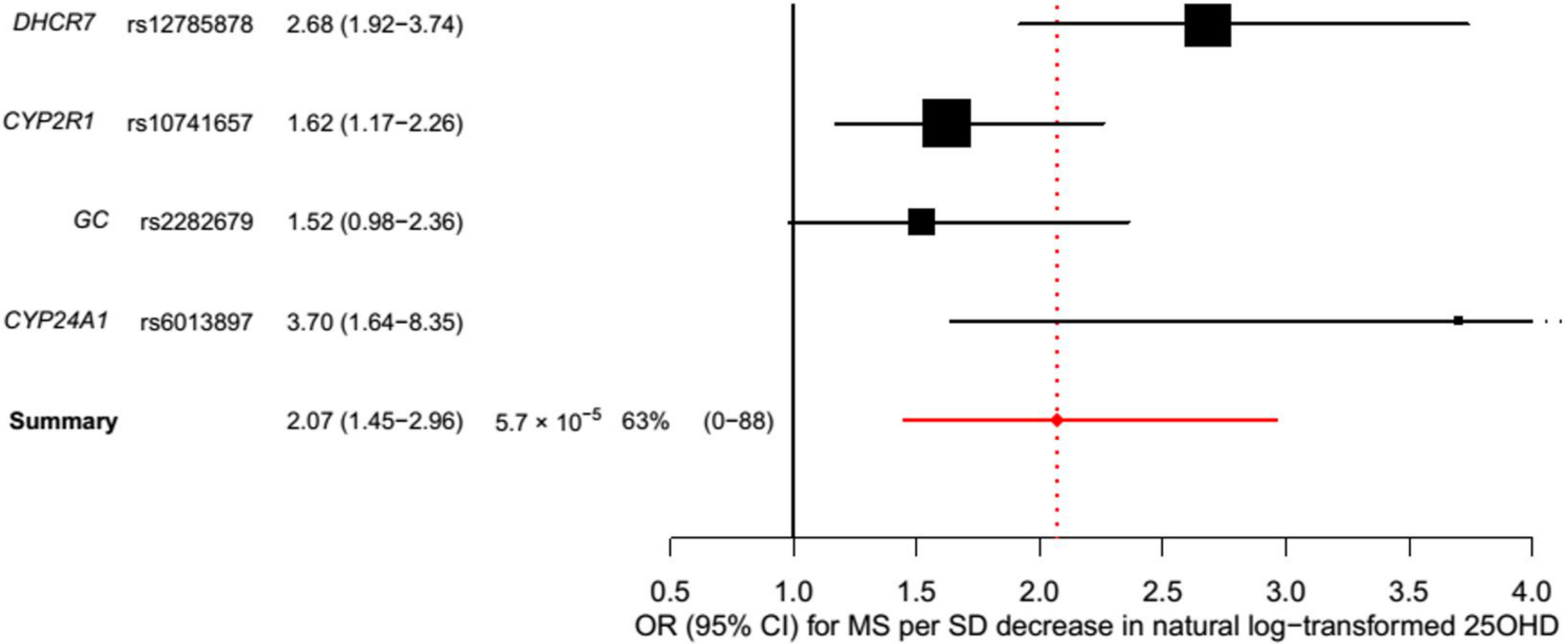

Supplement: S1 Fig — (PDF) [file pmed.1001866.s002.pdf]
