# Supplementary material for: Vitamin D and Risk of Multiple Sclerosis: A Mendelian Randomization Study
Source: PLoS Med. 2015 Aug 25;12(8):e1001866. doi: 10.1371/journal.pmed.1001866 (PMC4549308; doi:10.1371/journal.pmed.1001866)

Figure S6: Mendelian Randomization Estimates for the Association of 25OHD Levels with Risk of MS for SNPs involved in 25OHD Metabolism Using a Fixed Effects Model

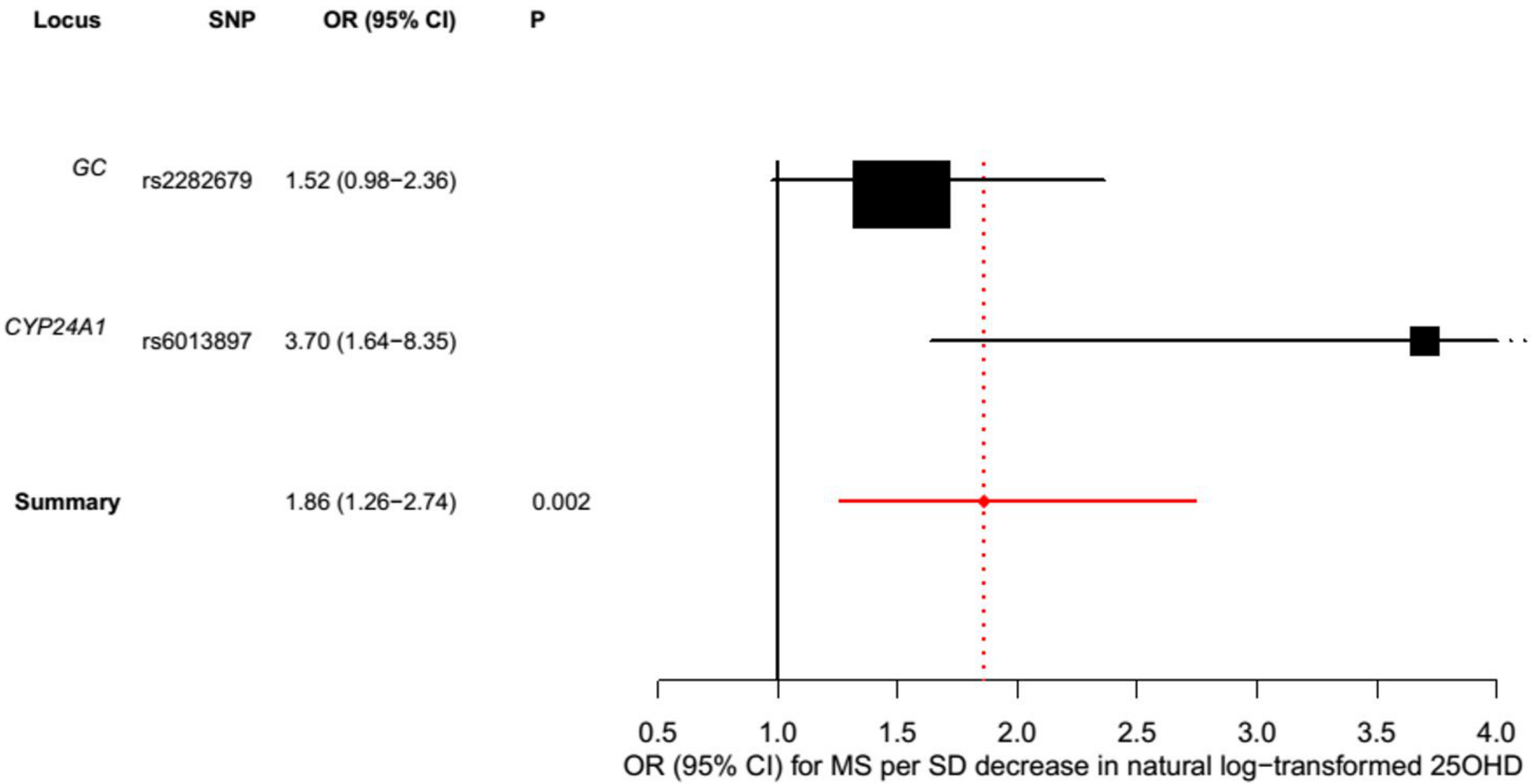

Supplement: S6 Fig — (PDF) [file pmed.1001866.s007.pdf]
