# Supplementary material for: Vitamin D and Risk of Multiple Sclerosis: A Mendelian Randomization Study
Source: PLoS Med. 2015 Aug 25;12(8):e1001866. doi: 10.1371/journal.pmed.1001866 (PMC4549308; doi:10.1371/journal.pmed.1001866)
Supplement: S1 Table — (PDF) [file pmed.1001866.s008.pdf]

Vitamin D and risk of Multiple Sclerosis: a Mendelian Randomization Study

Lauren E Mokry, Stephanie Ross, Omar S. Ahmad, Vincenzo Forgetta, George Davey-Smith, Aaron Leong, Celia MT Greenwood, George Thanassoulis, J. Brent Richards

Supplementary Table 1: Pleiotropy assessment

Summary Table for the association between our selected SNPs and other clinical traits (taken from previous work by Berry at al.) [1]

Biomarkers	P-Values			
	GC	*DHCR7*	*CYP24A1*	*CYP2R1*
vWF	0.43	0.30	0.97	0.10
tPA	0.51	0.17	0.27	0.34
D-dimer	0.80	0.66	0.63	0.65
Fibrinogen	0.66	0.90	0.61	0.40
CRP	0.04	0.44	0.08	0.99
Triglycerides	0.81	0.38	0.54	0.47
LDL	0.50	0.07	0.62	0.28
HDL	0.94	0.62	0.52	0.95
Cholesterol	0.73	0.10	0.70	0.39
FEV	0.56	0.95	0.76	0.20
Diastolic BP	0.17	0.34	0.065	0.12
Systolic BP	0.26	0.30	0.03	0.89
IgE	0.59	0.75	0.60	0.03
IGF-1	0.06	0.90	0.84	0.70
HbA1c	0.56	0.94	0.72	0.90

References:

1. Berry DJ, Vimaleswaran KS, Whittaker JC, Hingorani AD, Hyppönen E. Evaluation of genetic markers as instruments for Mendelian randomization studies on vitamin D. PLoS One. 2012;7. doi:10.1371/journal.pone.0037465
